# Impact of fellowship training for specialists on thyroidectomy outcomes of patients with thyroid cancer

**DOI:** 10.1038/s41598-024-59864-0

**Published:** 2024-04-19

**Authors:** Rujiao Lin, Sitao Huang, Xiumei Guo, Shengnan Gao, Feng Zheng, Zhengrong Zheng

**Affiliations:** 1 Department of Thyroid and Breast Surgery, The Second Affiliated Hospital of Fujian Medical University, Quanzhou, 362000 Fujian Province China; 2 Department of Neurosurgery, The Second Affiliated Hospital of Fujian Medical University, Quanzhou, 362000 Fujian Province China; 3 Department of Neurology, The Second Affiliated Hospital of Fujian Medical University, Quanzhou, 362000 Fujian Province China

**Keywords:** Thyroid cancer, Fellowship, Thyroid surgery, Prophylactic neck dissection, Postoperative complications, Impact, Health care, Health occupations, Medical research

## Abstract

We aimed to evaluate the impact of fellowship training (FT) for thyroid specialists on the outcomes of patients with thyroid cancer. We reviewed surgeries performed for thyroid cancer before (non-FT group) and after (FT group) fellowship training and compared several variables, including length of stay of patients, tumor diameter, surgical method, lymph node dissection, parathyroid implantation, surgical duration, intraoperative blood loss, and postoperative complications. Compared with the non-FT group, the FT group had a shorter hospital stay, more adequate fine needle aspiration biopsy of the thyroid, less intraoperative blood loss, higher rate of parathyroid implantation, higher lymph node dissection rate, and lower nerve injury and hypoparathyroidism rates. When the surgical duration was < 200 min and/or only central lymph node dissection was performed, the FT group had a lower incidence of postoperative complications than the non-FT group. When, the incidence of postoperative complications, including postoperative nerve injury and hypoparathyroidism. In conclusion, FT for thyroid specialists is beneficial for patients with thyroid cancer and may allow a shorter hospital stay and reduced incidence of postoperative complication. Accordingly, FT may facilitate a more appropriate surgical approach with a preoperative pathological diagnosis.

## Introduction

The annual incidence of thyroid cancer has been increasing worldwide^1,2^, reaching nearly 10.1 per 100,000 women and 3.1 per 100,000 men^3^. As the tumor size gradually increases, the compression of the surrounding tissue becomes more pronounced, resulting in symptoms such as dyspnea, dysphagia, hoarseness, and Horner’s syndrome^4–6^.

Fellowship training (FT) for specialists is an important aspect of postgraduate medical education and allows training of qualified clinical specialists^7^. Micah et al.^8^ reported that physicians trained through an endourology fellowship had a higher stone-free rate, lower complication and reoperation rates, and better postoperative follow-up outcomes. Further, Shabnam et al.^7^ found that trained gynecologists had a lower incidence of ureteral injury during laparoscopic hysterectomy. Similar conclusions have been reached in studies on orthopedics, with trained orthopedic surgeons showing significantly shorter surgical times, fluoroscopy usage, and traction times^9–11^.

However, the effectiveness of FT in thyroid surgery remains unclear^12^. Therefore, this retrospective study aimed to compare hospitalization data and postoperative complication rates in patients who underwent thyroid surgical interventions, in order to determine whether clinicians with FT could improve the prognosis of patients with thyroid cancer.

## Methods

FT was conducted at the Thyroid and Breast Surgery Department of the Second Affiliated Hospital of Fujian Medical University, where the annual surgical volume was over 2000 cases. FT began in July 2019, before which no surgeons received FT. Since July 2019, all thyroid surgeons at our department have undergone the two-year FT. Surgeons in the FT and non-FT group have similar level of education and academic qualifications.

### Data source and patient selection

This was a retrospective study of thyroid cancer surgery in the Second Affiliated Hospital of Fujian Medical University, including patients who underwent surgeries performed by thyroid surgeons without (January 2018–October 2018) and with (May 2022–September 2022) FT. Baseline patient information, admission time, discharge time, surgical records, and postoperative complications were collected from the hospital database; moreover, telephone follow-up interviews were conducted. The collected parameters included patient ID, sex, age, hospital stay length, fine-needle aspiration biopsy (FNA) of the thyroid, selected surgical method, lymph node dissection, parathyroid implantation, surgical duration, intraoperative blood loss, and postoperative complications. We used the TNM staging system for thyroid cancer developed by the American Joint Committee on Cancer^13^ to divide the tumors into ≤ 1, 1–2, 2–4, and > 4 cm categories. Subsequently, we compared the characteristics of thyroid cancer between the FT and non-FT groups. According to the recommendations of the 2015 American Thyroid Association Management Guidelines for Adult Patients with Thyroid Nodules and Differentiated Thyroid Cancer^14^, thyroid cancer surgeries were divided into four categories: (1) unilateral (+ isthmus) thyroidectomy; (2) subtotal or near-total thyroidectomy; (3) total thyroidectomy; and (4) others, including thyroidectomy with microwave ablation, lymph node dissection, biopsy, and isthmus resection. Lymph node dissection was divided into three categories: (1) no dissection, (2) central lymph node dissection (CLND), and (3) central and cervical lymph node dissection.

### Ethics

This study was conducted in accordance with the current version of the Declaration of Helsinki and good clinical practice guidelines^15^. This retrospective study was approved by the Medical Ethics Committee of the Second Affiliated Hospital of Fujian Medical University(No. 610, 2023), which waived the requirement for informed consent.

### Statistical analysis

Statistical analyses were performed using IBM SPSS Statistics v.26 (IBM Corp., Armonk, NY, USA). Fisher’s exact test and the chi-square test were used to identify between-group differences in clinical data. Normally distributed quantitative data are expressed as mean ± standard deviation $\left( {\overline{{\text{x}}} \pm s} \right)$ and converted into categorical variables for between-group comparison. Qualitative data are described by frequency, with between-group comparisons using the chi-square and Fisher’s exact tests. Statistical significance was set at *p* < 0.05.

## Results

**Figure 1:**
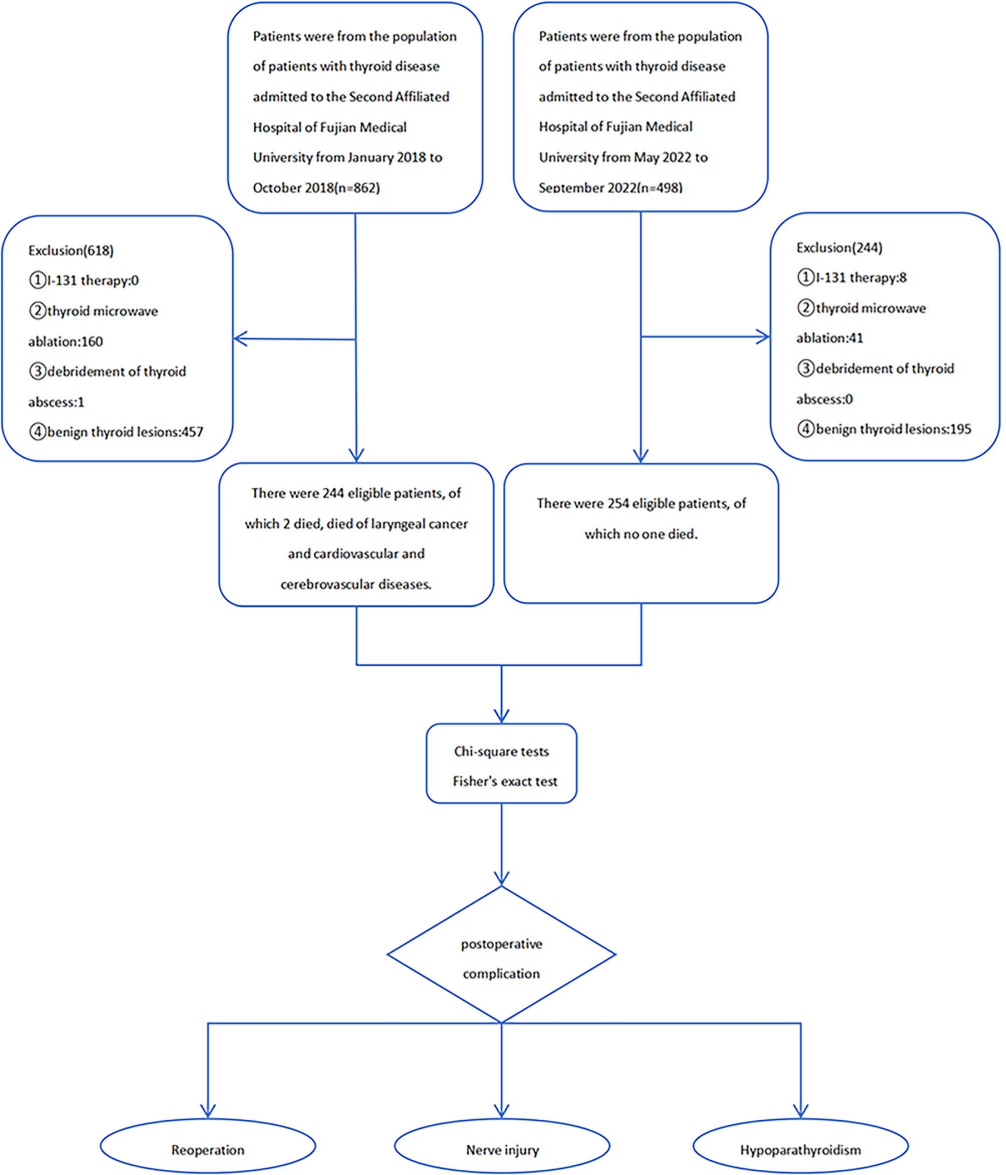
Flow chart of patient inclusion and exclusion.

Between January 2018 and October 2018 (non-FT group), 862 patients with thyroid disease were admitted to the Second Affiliated Hospital of Fujian Medical University. Among them, 618 patients were excluded, including 457 with benign thyroid diseases, 160 who underwent thyroid microwave ablation, and 1 with thyroid abscess debridement. Finally, we included 244 patients with thyroid cancer who underwent thyroidectomy. Between May 2022 and September 2022, 498 patients with thyroid diseases were admitted to the Second Affiliated Hospital of Fujian Medical University (FT group). Among them, 244 patients were excluded, including 195 with benign thyroid diseases, 41 with thyroid microwave ablation, and 8 with I^131^ therapy. Finally, we included 254 patients with thyroid cancer who underwent thyroidectomy. Although 17 and 7 patients in the non-FT and FT groups, respectively, were lost to follow-up, and 2 patients in the non-FT group died from other diseases after discharge, the hospital data for these patients were complete; therefore, they were included in the analysis. Ultimately, 498 patients with thyroid cancer were eligible for study inclusion (Fig. 1). Table 1 summarizes the clinical characteristics of the patients. All patients were followed up for ≥ 6 postoperative months.

**Table 1:** The demographic information and baseline characteristics of FT and non-FT group.

	FT	Non-FT	chi-square test	*P* value
Sex			0.692	0.405
Male	61 (24.0%)	51 (20.9%)		
Female	193 (76.0%)	193 (79.1%)		
Age	42.57 ± 12.00	43.19 ± 12.46	2.107	0.147
≤ 55	218 (85.8%)	200 (82.0%)		
> 55	36 (14.2%)	44 (18.0%)		
Tumor diameter(cm)	1.24 ± 1.08	1.48 ± 1.33	5.778	0.123
≤ 1	139 (54.7%)	123 (50.4%)		
1–2	75 (29.5%)	64 (26.2%)		
2–4	32 (12.6%)	40 (16.4%)		
> 4	8 (3.1%)	17 (7.0%)		
Hypertension	26 (27.5%)	28 (26.5%)	0.198	0.657
Diabetes	14 (11.7%)	9 (11.3%)	0.939	0.332
Coronary atherosclerotic heart disease	3 (2.6%)	2 (2.4%)	0.164	1.000
Hypothyroidism	3 (2.0%)	1 (2.0%)	0.929	0.624
Hyperthyroidism	9 (8.7%)	8 (8.3%)	0.026	1.000
Previous history of radiation	0 (0)	0 (0)	–	–
Family history	1 (0.4%)	0 (0)	0.963	1.000

Family history: The patient 's close relatives had thyroid cancer.

FT group: Surgical treatments were performed by trained thyroid physicians.

Non-FT group: Surgical treatments were performed by untrained thyroid physicians.

–: The data was not available.

### Impact of FT on hospitalization and surgical data

As shown in Table 2, compared with the non-FT group, the FT group had a shorter hospital stay (9.09 ± 5.207 vs. 5.38 ± 2.185 days, *p* = 0.000) and higher preoperative FNA rate (34.6 vs. 23.8%, *p* = 0.008). There were no significant between-group differences in the operating time (129.53 ± 65.960 [non-FT] vs. 117.75 ± 53.109 [FT] min, *p* = 0.095); however, the amount of intraoperative blood loss was lower in the FT group than in the non-FT group (20.08 ± 26.565 vs. 36.77 ± 86.097 mL, *p* = 0.000). Compared with the non-FT group, the FT group had a higher parathyroid implantation rate (66.9 vs. 11.1%, *p* = 0.000) and proportion of unilateral thyroidectomies (68.1 vs. 45.1%, *p* = 0.000).

**Table 2:** The comparison of the clinical features between FT and non-FT group.

	FT	Non-FT	chi-square test	*P* value
Length of hospital stay(days)	5.38 ± 2.185	9.09 ± 5.207	139.343	0.000*
≤ 5	166 (65.4%)	33 (13.5%)		
> 5	88 (34.6%)	211 (86.5%)		
Surgical methods			91.644	0.000*
Unilateral(+ isthmus) thyroidectomy	173 (68.1%)	110 (45.1%)		
Subtotal/near-total thyroidectomy	19 (7.5%)	84 (34.4%)		
Total thyroidectomy	62 (24.4%)	27 (11.1%)		
Others	0 (0)	23 (9.4%)		
Parathyroid implantation	170 (66.9%)	27 (11.1%)	162.434	0.000*
FNA	88 (34.6%)	58 (23.8%)	7.103	0.008*
Lymph node dissection			16.553	0.000*
Central lymph node dissection	218 (85.8%)	175 (71.7%)		
Central + cervical lymph node dissection	18 (7.1%)	25 (10.2%)		
No dissection	18 (7.1%)	44 (18.0%)		
Duration of surgery(min)	117.75 ± 53.109	129.53 ± 65.960	4.716	0.095
≤ 100	121 (47.6%)	96 (39.3%)		
100–200	116 (45.7%)	122 (50.0%)		
> 200	17 (6.7%)	26 (10.7%)		
Intraoperative blood loss(ml)	20.08 ± 26.565	36.77 ± 86.097	39.533	0.000*
≤ 30	233 (91.7%)	169 (69.3%)		
> 30	21 (8.3%)	74 (30.3%)		
Reoperation	1 (0.4%)	2 (0.8%)	0.377	0.617
Nerve injury	18 (7.3%)	38 (16.9%)	10.380	0.001*
Hypoparathyroidism	31 (12.6%)	56 (24.9%)	11.922	0.001*
Total	254	244		

*FNA*: fine needle aspiration biopsy of thyroid.

FT group: Surgical treatments were performed by trained thyroid physicians.

Non-FT group: Surgical treatments were performed by untrained thyroid physicians.

*: *p* < 0.05, indicates that the difference is statistically significant.

### Postoperative complications

There were two postoperative bleeding cases (0.8%) in the non-FT group and one (0.4%) in the FT group, with no significant between-group difference (*p* = 0.617). Compared with the non-FT group, the FT group had lower risks of postoperative nerve injury (7.3 vs. 16.9%, *p* = 0.001) and hypoparathyroidism (12.6 vs. 24.9%, *p* = 0.001) (Table 2).

As shown in Table 3, patients were further stratified according to their operating time. When surgical duration was ≤ 200 min, the risks of nerve injury (7.0 vs. 16.4%, *p* = 0.002) and hypoparathyroidism (12.6 vs. 25.4%, *p* = 0.001) were lower in the FT group than in the non-FT group, with no significant between-group difference in nerve injury (*p* = 0.679) and hypoparathyroidism (*p* = 0.679) when the operation time was > 200 min.

**Table 3:** The incidence of never injury and hypoparathyroidism based on different operation time and lymph node dissection ranges between FT and non-FT group.

	FT	Non-FT	chi-square test	*P* value
Duration of surgery ≤ 200 min				
Nerve injury	16 (7.0%)	33 (16.4%)	9.529	0.002*
Hypoparathyroidism	29 (12.6%)	51 (25.4%)	11.561	0.001*
> 200 min				
Nerve injury	2 (11.8%)	5 (20.8%)	0.578	0.679
Hypoparathyroidism	2 (11.8%)	5 (20.8%)	0.578	0.679
Not dissection				
Hypoparathyroidism	2 (11.8%)	11 (26.2%)	1.466	0.310
Nerve injury	1 (5.9%)	11 (26.2%)	3.080	0.150
Central lymph node dissection				
Hypoparathyroidism	28 (13.2%)	39 (24.1%)	7.374	0.007*
Nerve injury	15 (7.1%)	22 (13.6%)	4.359	0.037*
Central + cervical lymph node dissection				
Hypoparathyroidism	1 (5.6%)	6 (28.6%)	3.486	0.098
Nerve injury	2 (11.1%)	5 (23.8%)	1.061	0.418
Total	247	225		

FT group: Surgical treatments were performed by trained thyroid physicians.

Non-FT group: Surgical treatments were performed by untrained thyroid physicians.

*: *p* < 0.05, indicates that the difference is statistically significant.

Postoperative complications occurred at different ranges of lymph node dissection between the two groups. In patients with CLND, the incidences of nerve injury (7.1 vs. 13.6%, *p* = 0.037) and hypoparathyroidism (13.2 vs. 24.1%, *p* = 0.007) were lower in the FT group than in the non-FT group, with no significant between-group differences in the incidence of nerve injury (*p* = 0.150) or hypoparathyroidism (*p* = 0.310) among patients without lymph node dissection. In patients who underwent central and cervical lymph node dissection, there were no significant between-group differences in the incidence of nerve injury (*p* = 0.418) or hypoparathyroidism (*p* = 0.098).

## Discussion

Over the past three decades, there has been a gradual increase in the incidence of thyroid cancer, which may be attributed to its active detection and early diagnosis^16–19^. This growth poses a significant challenge for healthcare systems worldwide. Therefore, clinicians are required to actively improve clinical diagnosis and treatment techniques to delay thyroid cancer progression, and therefore improve patient outcomes^14^.

**Figure 2:**
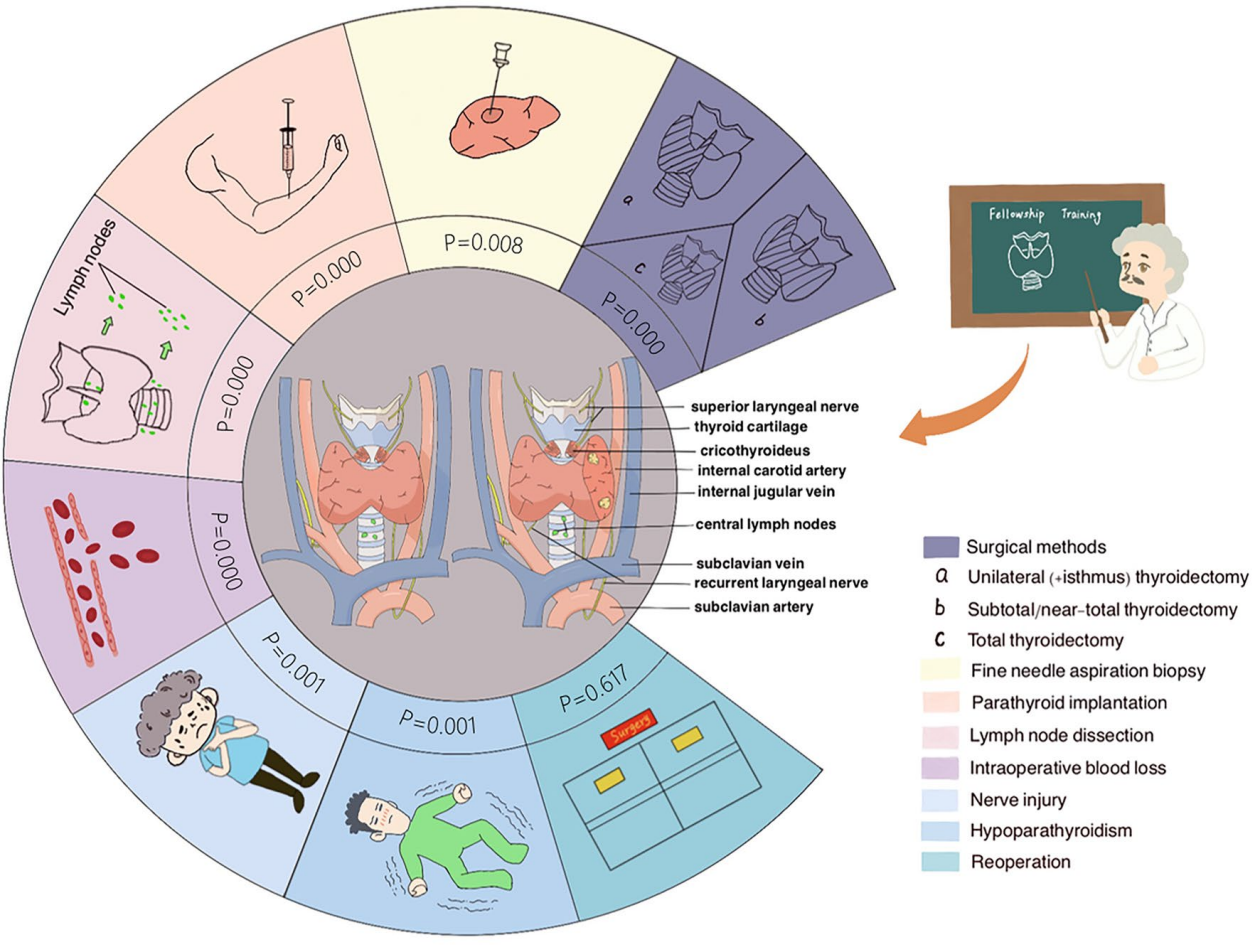
Comparison of complications between FT and non-FT groups. (Created by authors with the software of Procreate).

We have illustrated in Fig. 2 the similarities and differences in the extent of thyroidectomy, related surgical operations, and major surgical complications between the two groups. In our study, compared with the non-FT group, the FT group had a shorter hospital stay and less intraoperative blood loss. This indicated that patients in the FT group had less intraoperative injury and a faster postoperative recovery, subsequently leading to a reduction in hospitalization costs.

Preoperative FNA is helpful in the diagnosis of benign and malignant thyroid nodules in order to determine the optimal treatment and whether lymph node dissection is warranted^20^. The procedure helps determine the preoperative nature of the thyroid mass in order to allow patients to obtain optimal benefits from the smallest surgical range^21^. The preoperative FNA rate was higher in the FT group than in the non-FT group.

In this study, 45.1% and 68.1% of patients in the non-FT and FT groups, respectively, underwent a unilateral thyroidectomy. This could be attributed to trained thyroid physicians having better diagnostic skills and treatment techniques, which maximized benefits for patients with fewer injuries^22,23^. Furthermore, it may be related to the improvement in imaging technology^24^, which has contributed to the early diagnosis and treatment of patients with thyroid cancer. Compared with the non-FT group, the FT group had more patients who underwent unilateral thyroidectomy, which may explain the shorter average hospitalization time and fewer postoperative complications in this group. In our study, consistent with the American Thyroid Association Guidelines^14^, total thyroidectomy was implemented for patients who had distant metastases, marked extraglandular invasion, clear lymph node metastasis, poorly differentiated pathological subtypes, and high-risk factors for previous head and neck radiation exposure history. Patients who undergo total thyroidectomy are required to take thyroxine for life, which impacts their lives and finances. Therefore, patients in the FT group underwent more unilateral thyroidectomies within the scope of surgical indications.

In the FT group, 85.8% of patients underwent CLND, with this proportion being 14.1% higher than that in the non-FT group. Most patients had negative lymph nodes on both preoperative and postoperative pathology evaluations. Therefore, we opted for preventive dissection of the CLND (pCLND). However, it remains unclear whether pCLND should be performed in patients with cN0 thyroid cancer^25,26^. Therefore, it is important to thoroughly assess the specific situation of patients, evaluate the risk or benefit of pCLND, and make a choice that is the least harmful but also the most beneficial strategy for patients. In our study, only 7.1% of patients in the FT group did not undergo lymph node dissection, with the implementation rate being 10.9% lower than that in the non-FT group. This indicates that trained thyroid physicians are more inclined to perform pCLND, which allows accurate staging for predicting prognosis and determining subsequent treatment^20^.

The intraoperative parathyroid implantation rate was higher in the FT group than in the non-FT group, indicating a reduced risk of postoperative hypoparathyroidism in patients with thyroid cancer. In terms of the operation time, the incidence of hypoparathyroidism was lower in the FT group than in the non-FT group when the operation time was ≤ 200 min. Due to the small sample size of this study, this finding requires further verification. In terms of the extent of lymph node dissection, we observed a significant between-group difference in the incidence of nerve injury and hypoparathyroidism among patients undergoing central lymph node dissection. Specifically, the incidence of hypoparathyroidism in the FT group was 10.9% lower than that in the non-FT group, indicating that patients with thyroid cancer who were treated by trained thyroid physicians had a lower risk of postoperative hypoparathyroidism. There was no significant between-group difference in the incidence of nerve injury or hypoparathyroidism among patients who did not undergo lymph node dissection. This could be attributed to the fact that physicians in both groups could perform a simple thyroidectomy and avoid damage to the parathyroid gland, reducing the incidence of postoperative hypoparathyroidism. There was no significant between-group difference in the incidence of nerve injury and hypoparathyroidism among patients who underwent dissection of the central and lateral cervical lymph nodes. This could be attributed to the larger extent of lymph node dissection, which significantly increased the incidence of postoperative complications.

Nerve paralysis may occur due to the inexperience of the surgeon, tumor invasion and infiltration, compression by a large tumor, and surgical manipulation, including stretch, compression, thermal injury, or transection^27^. In the present study, the occurrence rate of postoperative nerve injury was lower in the FT group than that in the non-FT group, which may be explained by the fact that trained surgeons better understand the nerve anatomy, and thus avoid nerve damage. Accordingly, fewer patients in the FT group presented with postoperative symptoms, including hoarseness and coughing. With regard to operating time stratification, when the operating time was ≤ 200 min, the rate of occurrence of nerve injury was lower in the FT group than in the non-FT group. This may be due to the fact that trained thyroid surgeons can better grasp the anatomical structure around the thyroid gland as well as the critical points and difficulties of thyroid surgery, which allows avoidance of intraoperative nerve damage. These parameters did not show a significant between-group difference when the operating time was > 200 min. This may be related to the long-term traction of the tissue during surgery, which deserves more attention in clinical practice. Based on the extent of lymph node dissection, we found a significant between-group difference in the rate of occurrence of nerve injury and hypoparathyroidism among patients who underwent CLND. Specifically, the rate of occurrence of nerve injury was 6.5% lower in the FT group than in the non-FT group, indicating that trained thyroid physicians can reduce the incidence of postoperative nerve injury.

The present study had some limitations. First, this was a retrospective study; therefore, any conclusions drawn were subject to the limitations of the respective study design, including recall and observation bias. Second, the follow-up period in the non-FT group was longer than that in the FT group; accordingly, subacute and late-onset complications were more likely to be reported over a longer follow-up period. Third, this study was conducted at a single center with a restricted sample size. The relatively small sample size may have compromised the power of the primary results. Future studies are warranted to focus on this topic.

## Conclusions

Our findings showed that FT for thyroid specialists is beneficial for patients with thyroid cancer. Patients undergoing thyroidectomy by physicians with FT show reduced hospital stay, intraoperative blood loss, and postoperative complications, as well as increased rates of preoperative FNA, parathyroid implantation, and preventive central lymph node dissection. Future s large-scale studies are warranted to confirm our findings.

## Data Availability

The datasets used and/or analysed during the current study available from the corresponding author on reasonable request.

## References

[1] La Vecchia C, Malvezzi M, Bosetti C (2015). Thyroid cancer mortality and incidence: A global overview: Thyroid Cancer Mortality and Incidence. Int. J. Cancer.

[2] Vaccarella S, Dal Maso L, Laversanne M (2015). The impact of diagnostic changes on the rise in thyroid cancer incidence: A population-based study in selected high-resource countries. Thyroid.

[3] Pizzato M, Li M, Vignat J (2022). The epidemiological landscape of thyroid cancer worldwide: GLOBOCAN estimates for incidence and mortality rates in 2020. Lancet Diabetes Endocrinol..

[4] Broome JT, Gauger PG, Miller BS (2009). Anaplastic thyroid cancer manifesting as new-onset horner syndrome. Endocr. Pract..

[5] Collazo-Clavell ML, Gharib H, Maragos NE (1995). Relationship between vocal cord paralysis and benign thyroid disease. Head Neck.

[6] Cipriani NA, White MG, Angelos P (2018). Large cytologically benign thyroid nodules do not have high rates of malignancy or false-negative rates and clinical observation should be considered: A meta-analysis. Thyroid.

[7] Gupta S, Maghsoudlou P, Ajao M (2022). Very low rates of ureteral injury in laparoscopic hysterectomy performed by fellowship-trained minimally invasive gynecologic surgeons. J. Minim. Invasive Gynecol..

[8] Levy M, Chin CP, Walt A (2023). The role of experience: how case volume and endourology-fellowship training impact surgical outcomes for ureteroscopy. J. Endourol..

[9] Fisher BT, Chong ACM, Flick T (2021). Does surgeon subspecialty training affect outcomes in the treatment of displaced supracondylar humerus fractures in children?. J. Am. Acad. Orthop. Surg..

[10] Levy HA, Karamian BA, Yalla GR (2022). Effect of fellow involvement and experience on patient outcomes in spine surgery. J. Am. Acad. Orthop. Surg..

[11] Flores SE, Borak KR, Zhang AL (2018). Hip arthroscopic surgery for femoroacetabular impingement: A prospective analysis of the relationship between surgeon experience and patient outcomes. Orthop. J. Sports Med..

[12] Harness JK, Van Heerden JA, Lennquist S (2000). Future of thyroid surgery and training surgeons to meet the expectations of 2000 and beyond. World J. Surg..

[13] Perrier ND, Brierley JD, Tuttle RM (2018). Differentiated and anaplastic thyroid carcinoma: Major changes in the American joint committee on cancer eighth edition cancer staging manual. CA Cancer J. Clin..

[14] Haugen BR, Alexander EK, Bible KC (2016). 2015 American thyroid association management guidelines for adult patients with thyroid nodules and differentiated thyroid cancer: The American thyroid association guidelines task force on thyroid nodules and differentiated thyroid cancer. Thyroid.

[15] Parsa-Parsi RW (2022). The international code of medical ethics of the world medical association. JAMA.

[16] Schuster-Bruce J, Jani C, Goodall R (2022). A comparison of the burden of thyroid cancer among the european union 15+ countries, 1990–2019: Estimates from the global burden of disease study. JAMA Otolaryngol. Head Neck Surg..

[17] Lim H, Devesa SS, Sosa JA (2017). Trends in thyroid cancer incidence and mortality in the United States, 1974–2013. JAMA.

[18] Wu J, Zhao X, Sun J (2022). The epidemic of thyroid cancer in China: Current trends and future prediction. Front. Oncol..

[19] Li Y, Piao J, Li M (2021). Secular trends in the epidemiologic patterns of thyroid cancer in China over three decades: An updated systematic analysis of global burden of disease study 2019 Data[J]. Front. Endocrinol..

[20] Feng J-W, Pan H, Wang L (2019). Determine the optimal extent of thyroidectomy and lymphadenectomy for patients with papillary thyroid microcarcinoma. Front. Endocrinol..

[21] Gharib H (2018). Thyroid Nodules.

[22] Zhao H, Cui L (2021). Extent of surgery and the prognosis of unilateral papillary thyroid microcarcinoma. Front. Endocrinol..

[23] Gartland RM, Lubitz CC (2018). Impact of extent of surgery on tumor recurrence and survival for papillary thyroid cancer patients. Ann. Surg. Oncol..

[24] Nabhan F, Dedhia PH, Ringel MD (2021). Thyroid cancer, recent advances in diagnosis and therapy. Int. J. Cancer.

[25] Zhao W, Luo H, Zhou Y (2017). Evaluating the effectiveness of prophylactic central neck dissection with total thyroidectomy for cN0 papillary thyroid carcinoma: An updated meta-analysis. Eur. J. Surg. Oncol..

[26] Ahn S-H, Kim WS (2020). The effect of prophylactic central neck dissection during hemithyroidectomy on locoregional recurrence in patients with papillary thyroid carcinoma: A meta-analysis. Clin. Exp. Otorhinolaryngol..

[27] Russell MD, Kamani D, Randolph GW (2019). Surgical management of the compromised recurrent laryngeal nerve in thyroid cancer. Best Pract. Res. Clin. Endocrinol. Metab..

